# Antibodies as biomarker candidates for response and survival to checkpoint inhibitors in melanoma patients

**DOI:** 10.1186/s40425-019-0523-2

**Published:** 2019-02-20

**Authors:** Mirjam Fässler, Stefan Diem, Joanna Mangana, Omar Hasan Ali, Fiamma Berner, David Bomze, Sandra Ring, Rebekka Niederer, Cristina del Carmen Gil Cruz, Christian Ivan Pérez Shibayama, Michal Krolik, Marco Siano, Markus Joerger, Mike Recher, Lorenz Risch, Sabine Güsewell, Martin Risch, Daniel E. Speiser, Burkhard Ludewig, Mitchell P. Levesque, Reinhard Dummer, Lukas Flatz

**Affiliations:** 1 Institute of Immunobiology, Kantonsspital St.Gallen, Rorschacherstrasse 95, 9007 St. Gallen, Switzerland; 2 Department of Dermatology, Allergology and Venerology, Kantonsspital St.Gallen, Rorschacher Str. 95, 9007 St. Gallen, Switzerland; 3 Department of Oncology/Hematology, Kantonsspital St.Gallen, Rorschacher Str. 95, 9007 St. Gallen, Switzerland; 4 Department of Oncology/Hematology, Spital Grabs, Spitalstrasse 44, 9472 Grabs, Switzerland; 5 Department of Dermatology, University Hospital Zurich, University of Zurich, Rämistrasse 100, 8091 Zurich, Switzerland; 6 Clinic for Primary Immunodeficiency, Medical Outpatient Unit and Immunodeficiency Laboratory, Department of Biomedicine, University Hospital, Hebelstrasse 20, 4067 Basel, Switzerland; 7 Labormedizinisches Zentrum Dr. Risch Ostschweiz AG, Brauerstrasse 95, 9016 St. Gallen, Switzerland; 8 Center of Laboratory Medicine, University Institute of Clinical Chemistry, University of Bern, Inselspital, INO-F, 3010 Bern, Switzerland; 9 Private University Triesen, Dorfstrasse 24, 9495 Triesen, Liechtenstein; 10 Clinical Trials Unit, Kantonsspital St.Gallen, Bedastrasse 1, 9000 St. Gallen, Switzerland; 11 Department of Laboratory Medicine, Kantonsspital Graubünden, Loestrasse 170, 7000 Chur, Switzerland; 12 Ludwig Cancer Research, University of Lausanne, Chemin des Boveresses 155, 1066 Épalinges, Lausanne, Switzerland

**Keywords:** Metastatic melanoma, Checkpoint inhibitors, Biomarker, Immune response, Antibodies, Melanocyte differentiation antigens, Cancer/testis antigens, gp100, TRP1, TRP2, MART1, NY-ESO-1

## Abstract

**Background:**

Long-term survival of stage IV melanoma patients has improved significantly with the development of immune checkpoint inhibitors (CIs). Reliable biomarkers to predict response and clinical outcome are needed.

**Methods:**

We investigated the role of melanoma-associated antibodies as predictive markers for CI therapy in two independent cohorts. In cohort 1, a prospective study, we measured specific antibodies before treatment, after one week and after six to nine weeks of treatment. Cohort 2 consisted of serum samples prior to CI therapy initiation. ELISA assays were performed to quantify specific IgG directed against melanocyte differentiation antigens tyrosinase-related proteins 1 and 2 (TRP1/TYRP1 and TRP2/TYRP2), glycoprotein 100 (gp100), MelanA/MART1, and the cancer-testis antigen NY-ESO-1. Response was defined as either complete or partial remission on CT scan according to RECIST 1.1.

**Results:**

In cohort 1, baseline levels of these antibodies were higher in the responder group, although statistical significance was only reached for NY-ESO-1 (*p* = 0.007). In cohort 2, significantly higher antibody baseline levels for MelanA/MART1 (*p* = 0.003) and gp100 (*p* = 0.029) were found. After pooling the results from both cohorts, higher levels of MelanA/MART1 (*p* = 0.013), TRP1/TYRP1 (*p* = 0.048), TRP2/TYRP2 (*p* = 0.047) and NY-ESO-1 (*p* = 0.005) specific antibodies at baseline were independently associated with response.

**Conclusions:**

Melanoma-associated antibodies may be candidate biomarkers for response and survival in metastatic melanoma patients being treated with CIs. These markers may be used to complement patient assessment, in combination with PD-L1 status, tumor-infiltrating lymphocytes and tumor mutational burden, with the aim to predict outcome of CI treatment in patients with metastatic melanoma.

**Trial registration:**

Ethikkommission Ostschweiz, EKOS 16/079 https://ongoingprojects.swissethics.ch/runningProjects_list.php?q=%28BASECID~contains~2016-00998%29&orderby=dBASECID.

**Electronic supplementary material:**

The online version of this article (10.1186/s40425-019-0523-2) contains supplementary material, which is available to authorized users.

## Background

Survival of patients suffering from metastatic melanoma has significantly improved since the introduction of immune checkpoint inhibitors (CIs). CIs activate the immune system by blocking inhibitory signals between T cells and tumor cells or antigen-presenting cells. The cytotoxic-T-lymphocyte-associated-protein-4 (CTLA4) targeting antibody ipilimumab was the first clinically approved CI, with a significantly increased response rate compared to previous treatments and a survival rate of about 20% after 10 years in patients with advanced melanoma [1–3]. The anti-programmed-cell-death-protein-1 (PD1) antibodies nivolumab and pembrolizumab show response rates of around 40% as single agents, and improved progression free survival (PFS) and overall survival (OS) compared to chemotherapy or ipilimumab [4–7]. Response rates can rise up to 60% when anti-PD1 therapy is combined with anti-CTLA4 [8, 9]. However, not all patients respond to CI treatment. Furthermore, patients are at risk of developing immune-related adverse events (irAEs) including colitis, pneumonitis and endocrine abnormalities. While irAEs are manageable in most patients, fatal cases have been reported [1].

Melanoma is known to be one of the most immunogenic tumors, as underlined by several observations including frequency of spontaneous tumor regression and higher prevalence of melanoma in immunosuppressed individuals, indicating that immunosurveillance plays a key role in melanoma [10–12]. PD-L1 expression, pre-treatment tumor infiltrating lymphocytes (TILs), lactate dehydrogenase (LDH) and hematological parameters including absolute lymphocyte count have been evaluated as predictive markers for CI therapy [8, 13–18]. However, many of these markers remain difficult to implement in routine diagnostics [19]. Many associations (e.g.: PD-L1 expression on tumor cells) have been shown to correlate with CI therapy outcome. To predict responses to treatment, Blank and colleagues proposed a “cancer immunogram” that integrates seven parameters consisting of general immune status, immune cell infiltration, PD-L1 expression, absence of soluble immune inhibitors, absence of inhibitory tumor metabolism, tumor sensitivity to immune effectors and tumor foreignness. However, the ideal combination of parameters for a cancer immunogram able to predict responses to CI treatment is still unknown [20].

Tumor specific antibodies have been studied extensively over many years. Untreated patients suffering from primary and metastatic melanoma show higher levels of antibodies specific for melanocyte differentiation antigens (MDAs) and cancer-testis antigens as compared to healthy volunteers [21–23]. Pre-treatment levels of MDA-specific antibodies were found to correlate with clinical outcome in melanoma patients treated with various therapies, in times when CIs were not yet available for cancer patients [24, 25]. Recently, it was shown that ipilimumab enhances humoral immunity against NY-ESO-1 and that this antibody response is associated with a clinical benefit to ipilimumab treatment [26]. Based on these findings, we hypothesized that pre-existing antibodies against a broader range of antigens may correlate with clinical outcome of melanoma patients treated with therapies targeting PD-1/PD-L1 and CTLA4.

## Methods

### Patient cohort

Cohort 1 consisted of prospectively collected clinical and laboratory data from patients with metastatic melanoma at the Kantonsspital St. Gallen (Switzerland), starting treatment with anti-PD1 or anti-CTLA4 antibodies between August 2016 and March 2017.

Patients had at least two treatment cycles of either nivolumab (Opdivo; Bristol-Myers Squibb SA, 3 mg/kg every two weeks), pembrolizumab (Keytruda; MSD Merck Sharp & Dohme AG, 2 mg/kg every three weeks), ipilimumab (Yervoy; Bristol-Myers Squibb SA, 3 mg/kg every three weeks) or the combination of nivolumab and ipilimumab (1 mg/kg and 3 mg/kg every three weeks). Blood samples were collected at three time points: before treatment initiation, one week after the first administration of therapy and at the fourth cycle six to nine weeks after the first administration or in patients with less cycles at the last administration. Computed tomography (CT) scans were performed before the fourth cycle for evaluation of response to therapy and assessed according to RECIST 1.1 criteria [27]. Patients who showed progressive disease (PD) at the first CT-scan underwent another scan within four to six weeks to confirm PD and rule out pseudoprogression [28]. Response was defined as either complete remission (CR) or partial remission (PR). Non-responders were defined as patients showing stable disease (SD) or PD.

A group of eight patients (four responders and four non-responders) with advanced non-small-cell-lung-cancer (NSCLC) receiving CI therapy served as a control group for the enzyme-linked immunosorbent assay (ELISA) experiments. The examined patient cohort included exclusively Caucasians.

Cohort 2 was provided by the biobank of the Department of Dermatology of the University of Zurich (Switzerland). Serum pre-treatment samples from 21 patients suffering from stage IV melanoma treated with CI therapy were included.

Both study cohorts were approved by the local ethics committees (EKOS 16/079 respectively EK 647, EK800), and partly funded by the University Research Priority Program (URPP). Both studies were carried out in accordance with the Declaration of Helsinki principles.

### Selection of antigens

Two classes of non-mutated antigens are relatively frequently recognized by TILs in melanoma [29]. Firstly, antigens derived from MDAs, especially MelanA/MART1 and glycoprotein 100 (gp100), but also tyrosinase and tyrosinase-related proteins 1 (TRP1/TYRP1) and 2 (TRP2/TYPR2) have been characterized [30–34]. The fact that T cells specific for these antigens are abundantly present in TILs of melanoma patients indicates that these T cells undergo antigen-specific expansion. Furthermore, autoimmune toxicities, such as skin rashes, vitiligo and uveitis can occur in these patients, likely as a result of melanocyte destruction [35, 36].

A second class of antigens recognized by melanoma TILs are cancer/testis (C/T) antigens. Such antigens, including the MAGE family of antigens, SSX2, NY-ESO-1, RAGE and SAGE were discovered within the last decades [37–39]. These antigens are usually expressed during embryogenesis and in germ cells, and silenced in other adult tissues. However, it has been shown that tumors can abnormally express these genes.

In melanoma and other tumor types, beside antigen-specific T cells, also the importance of B cells has been increasingly reported [40–43]. Furthermore, the density of B cell infiltration was found to correlate with T cell activation, possibly implying a role for B cells in the activation of antitumor immune responses [44].

Based on these findings, we focused on five antigens for our investigation: gp100, MelanA/MART1, TRP1/TYRP1, TRP2/TYPR2 and NY-ESO-1.

### Detection of antibodies against melanoma self-antigens by ELISA

High-binding, 96-well clear polystyrene flat bottom plates (Corning, NY, USA) were coated overnight at 4 °C with recombinant full length human melanoma gp100 (Abcam, ab132146), MelanA/MART1 (Abcam, ab114312), TRP1/TYRP1 (Abcam, ab132102), NY-ESO-1 (LSBio, LS-G22876) or the N-Terminus portion amino acids 1 to 519 from TRP2/TYRP2 (Abcam, ab158268) dissolved in 0.1 M carbonate buffer (pH 9.5) (See Additional file 1: Table S1). Non-specific binding was blocked by incubating the plates 2 h at room temperature with 5% non-fat dry milk in phosphate buffered saline (PBS) pH 7.2. Patient sera were diluted in 5% non-fat dry milk-PBS immediately before use and incubated 2 h at room temperature. For detection peroxidase-conjugated anti-human IgG (Jackson ImmunoResearch, 109–035-003) was used in a 1:2′500 dilution and incubated 2 h at room temperature. The plates were developed with 0.5 mg/mL ortho-phenlyenediamine (Sigma, MO, USA) in 0.1 M citrate buffer (pH 5.6), containing 0.08% H_2_O_2_ (Sigma, MO, USA). The reaction was stopped with 1.25 M H_2_SO_4_, and the optical densities were read at 492 nm using an automatic ELISA plate reader (Tecan, Sunrise™, Switzerland) [3, 45, 46].

Assay robustness was established previously before evaluating the patient’s samples by altering experimental parameters (incubation times, coating concentration, serum and antibody dilutions). Two patient or volunteer samples with the highest and lowest signal in a test experiment were used to evaluate the optimal coating antigen concentration and serum dilution.

The optimal antigen coating concentrations and sera dilutions for each antigen were determined by performing checkerboard titration with 4 log2 dilutions of the antigen and 5 log2 dilutions of the sera (Additional file 1: Table S1). To determine the background signal in the ELISA for each antigen, five wells were left without serum. The mean absorbance of these wells plus three times their standard deviations was subtracted from all other absorbance values. The background signal represents non-specific binding and false positive results of the methodology. All ELISAs were performed in duplicates, using the mean values for analysis.

For the detection of antibodies against Epstein-Barr virus (EBV) commercially available ELISA kits (Medac Diagnostika, Wedel, Germany) were used.

For the anti-EBV EBNA-1-IgG detection, the patient sera were diluted 1:200 and mixed with the prepared conjugate from the kit. An amount of 50ul/well of this mix was added to the pre-coated plate and incubated at 37 °C in a humid box for 60 min. After intense washing, 50ul of the IgG conjugate was added and incubated at 37 °C in a humid box for 60 min. Following other wa shing cycles, 50ul of the TMB-substrate was added and incubated at 37 °C in a humid dark box for 30 min. The reaction was stopped with 0.5 M H_2_SO_4_, and the absorbance read at 450 nm using an automatic ELISA plate reader. EBNA-1-IgG levels were calculated and analyzed according to their instruction guidelines.

Specific IgG antibody titers against Varicella zoster virus (VZV) were measured by a commercially available fully automated method for quantitative antibody detection employing Chemiluminescence Immunoassay (CLIA) as a measurement principle (Diasorin Liaison VZV IgG, measured on a Liaison XL analyzer; Diasorin, Lucerne, Switzerland). With positive controls, the coefficient of variations (CV) in our hands was 6.86% at a mean antibody titer of 485 mIU/mL, and 5.77% at a mean antibody titer of 2154 mIU/mL. A titer of > 164 mIU/mL is considered a specific antibody response.

### Analyses of immunoglobulins

Total immunoglobulin was determined using a BN II nephelometer (Siemens Diagnostics, Zurich, Switzerland) using reagents from Siemens (Siemens Diagnostics, Zurich, Switzerland). In our hands, the imprecision of the employed methods, as assessed by CV obtained from serial measurements of commercially available control materials was as follows: 3% for total IgG (at concentrations of 7.1 and 13.2 g/L). The CV for the IgG was 4.0% (at a concentration of 4.63 g/L).

### Immunohistochemistry

Tissue samples prior to therapy were available for 9 out of 20 metastatic melanoma patients from cohort one. The samples were taken for diagnostic histological examination and were formalin-fixed and paraffin-embedded in the Department of Pathology of the Kantonsspital St. Gallen using the standard processing protocols. Four-micron-thick serial sections were then cut using a rotary microtome. Single epitope enzymatic immunohistochemistry on FFPE tissue was performed on serial sections to assess the % of tumor tissue expressing gp100 and MelanA/MART1 using a Leica BOND MAX automated immunostainer and the following antibodies: monoclonal mouse anti-human MelanA (Dako, catalog number M7196, clone A103, dilution 1:150, HIER - pH 9/20 min/95 °C, incubation for 15 min), and monoclonal mouse anti-human Melanosome (Dako, catalog number M0634, clone HMB-45, dilution 1:100, HIER - pH 6/20 min/100 °C, incubation for 30 min). Ten high power fields (HPF) equally distributed within the tumor were acquired from each case using a Leica DM RA microscope equipped with a Leica DFC420 C digital camera and processed using the Leica Application Suite version 3.8.0 (Leica Microsystems, Switzerland). Quantitative morphometry was performed using the ImageJ public domain Java image processing program as described in the supporting methods [47].

### Statistical analyses

Statistical analysis was performed separately for the two cohorts, and results were compared qualitatively.

Differences in serum IgG levels (both antigen-specific and total) between responders and non-responders before treatment start as well as changes during treatment in cohort 1 were illustrated by plotting the distribution of values per patient group. The significance of differences between responders and non-responders was tested using Wilcoxon rank-sum tests. Responders and non-responders were also compared within the control (NSCLC) group from cohort 1 with separate Wilcoxon rank-sum tests. The significance of changes during the course of treatment was analyzed with Friedman tests for each patient group in cohort 1. To test whether the change in IgG level differed between responders and non-responders, the difference between absorbance values at visits 1 and 5 was calculated for each patient, and differences were compared between groups with Wilcoxon rank sum tests.

The association between melanoma-associated IgG levels and either OS or PFS was examined with Kaplan-Meier survival curves drawn separately for responders and non-responders, as well as patients with high or low IgG baseline levels. High- and low-IgG groups were defined separately for each antigen by cutpoints maximizing the sum of sensitivity and specificity for the prediction of the response to CI therapies in a receiver operating characteristic (ROC) analysis. Differences in survival between patient groups were further analyzed using Cox proportional hazards regression models, and their significance was assessed using the log-rank test.

In order to test the association between response and melanoma-associated antibody levels for both cohorts together and with a simple approach that could be applied easily in clinical practice, we merged data from the two cohorts and classified all absorbance values into the three different groups (“strong”, “weak” and “no response detected”) by comparison with the mean value of the control (NSCLC) group from cohort 1. This mean was taken as cutpoint for a weak positive signal and its double as cutpoint for a strong positive signal. Relative frequencies of the three groups were compared between responders and non-responders using Fisher’s exact tests.

Changes over time in anti-EBV EBNA-1-IgG titers in responders and non-responders were tested with paired and differences in anti-EBV EBNA-1-IgG and anti-VZV-IgG titers between the two groups with unpaired t-tests.

Given the explorative nature of this study, *p*-values were not adjusted for multiple testing. Thus, “significant” differences reported here should be taken as indications of effects that warrant further testing, rather than results of formal hypothesis tests.

Correlation analysis to examine the relation between IgG levels specific for gp100 and MelanA/MART1 in serum and their corresponding antigen expression in tumor tissue was assessed using the Pearson and Spearman correlation tests after checking for normal distribution with Saphiro-Wilk.

Statistical analyses were performed using the software R, version 3.3.3. (R core Team 2017) or GraphPad Prism software version 7.0 [48]. The figures were then adjusted in Corel Draw Graphics Suite X8.

## Results

In the first cohort, 15 (75%) patients received monotherapy with an anti-PD1 antibody (nivolumab or pembrolizumab), three patients (15%) were treated with the combination of nivolumab plus ipilimumab and two patients (10%) with ipilimumab monotherapy. At the first CT scan performed after 9–12 weeks of therapy, one patient had a CR (5%), nine had a PR (45%), six showed SD (30%) and four patients had PD (20%). Two patients from the SD group initially had a pseudoprogression as they presented with partial response at the second scan. In summary, the first cohort consisted of 60% [12] responders showing CR/PR and of 40% [8] non-responders (SD, PD). For more information see patient characteristics in Table 1.

**Table 1:** Patient characteristics and outcome, cohort 1

Patient	Response	Characteristics (m^1^/f^2^; age (y^3^))	Phototype	Histological type	BRAF Status (wt^5^/mut^6^)	Checkpoint inhibitor therapy	Number of involved organs	Metastasis	ECOG^9^ Performance status	Tumor Response at first CT scan^10^
1	Responders	m, 70	2	SSM^4^	mut	anti-PD1^7^	2	Lung, Lymph nodes	0	PR
2		m, 70	3	SSM	wt	anti-PD1	3	Soft tissue, Bone, Liver	0–1	SD*
3		f, 78	2	SSM	wt	anti-PD1 + anti-CTLA4^8^	3	Soft tissue, Lymph nodes, Lung	0	PR
4		m, 63	3	SSM	wt	anti-PD1	1	Lung	0	PR
5		m, 52	3	SSM	mut	anti-PD1 + anti-CTLA4	4	Mesenterium, Peritoneum, Retroperitoneum, Brain	0	SD*
6		m, 86	3	nodular	wt	anti-PD1	2	Bone, Lung	0	PR
7		f, 66	2	nodular	mut	anti-CTLA4	5	Lymph nodes, Lung, Soft tissue, Suprarenal gland, Stomach	0	PR
8		f, 81	2	nodular	wt	anti-PD1	5	Lymph node, Soft tissue, Lung, Bone, Liver	0	PR
9		f, 66	2	nodular	wt	anti-PD1	3	Soft tissue, Lymph nodes, Brain	0	PR
10		m, 78	2	nodular	wt	anti-PD1	3	Soft Tissue, Lymph nodes, Lung	0	CR
11		f, 61	1	uveal	wt	anti-PD1	1	Bone	0	PR
12		m, 66	2	mucosal	wt	anti-PD1	6	Soft tissue, Lung, Pankreas, Small pelvis, Liver, Bone	0	PR
13	Non-Responders	m, 62	2	SSM	wt	anti-PD1	4	Suprarenal gland, Lung, Lymph node, Brain	0	SD
14		f, 56	2	SSM	mut	anti-PD1 + anti-CTLA4	5	Lung, Lymph node, Soft tissue, Liver, Stomach	1	PD
15		f, 87	3	nodular	mut	anti-PD1	5	Lung, Lymph node, Liver, Bone, Brain	1	SD
16		f, 71	2	uveal	wt	anti-CTLA4	3	Lung, Liver, Brain	0	PD
17		f, 71	2	uveal	wt	anti-PD1	2	Liver, Lymph node	0	SD
18		f, 87	2	mucuosal	wt	anti-PD1	1	Soft tissue	1	PD
19		f, 71	2	unknown, amelanotic	wt	anti-PD1	3	Lung, Lymph node, Suprarenal gland	0	SD
20		m, 72	3	unknown	wt	anti-PD1	7	Lung, Liver, Lymph node, Suprarenal gland, Pankreas, Bone, Eye muscle	0	PD

* pseudoprogression, ^1^ male, ^2^ female, ^3^ years, ^4^ superficial spreading melanoma, ^5^ wild type, ^6^ V600E mutation, ^7^ anti-programmed-cell-death protein-1, ^8^anti-cytotoxic-T-lymphocyte-associated-protein-4, ^9^Eastern Cooperative Oncology Group,^10^ *CR* Complete Remission, *PR* Partial Remission, *SD* Stable Disease, *PD* Progressive Disease

In cohort two, 18 (86%) patients were treated with anti-PD1 monotherapy, while the other three (14%) patients underwent the combination therapy (nivolumab plus ipilimumab). 11 of the patients showed a PR (52%) at the first CT scan and four patients had SD (19%). All patients with an initial pseudoprogression showed a partial remission in an additional CT scan performed 4–6 weeks later leading to 71% [15] of responders and 29% [6] of non-responders (Table 2).

**Table 2:** Patient characteristics and outcome, cohort 2

Patient	Response	Characteristics (m^1^/f^2^; age (y^3^))	Phototype	Histological type	BRAF Status (wt^7^/mut^8^)	Checkpoint inhibitor therapy	Number of involved organs	Metastasis	Tumor Response at first CT scan^11^
1	Responders	f, 35	n.a.^4^	SSM^5^	mut	anti-PD1^9^ + anti-CTLA4^10^	4	Soft tissue, Lung, Liver, Spleen	PR
2		m, 93	2	SSM	wt	anti-PD1	2	Lymph nodes, Lung	SD^*^
3		f, 49	2	SSM	mut	anti-PD1 + anti-CTLA4	4	Lung, Liver, Lymph nodes, Brain	PR
4		f, 43	3	SSM	wt	anti-PD1	4	Lung, Lymph nodes, Soft tissue, Brain	PR
5		f, 54	2	SSM	mut	anti-PD1	2	Soft tissue, Lymph nodes	PR
6		m, 48	n.a.	nodular	wt	anti-PD1	3	Lymph nodes, Soft tissue, Bone	PR
7		m, 57	2	nodular	wt	anti-PD1	7	Soft tissue, Lymph nodes, Kidney, Peritoneum, Lung, Bone, Brain	SD^*^
8		f, 53	2	nodular	mut	anti-PD1	1	Lung	SD^*^
9		m, 36	2	nodular	wt	anti-PD1	2	Lung, Lymph nodes	PR
10		m, 75	n.a.	nodular	wt	anti-PD1	1	Lung	PR
11		m, 69	2	nodular	wt	anti-PD1	1	Soft tissue	PR
12		f, 49	2	nodular	wt	anti-PD1	1	Lung	PR
13		m, 30	4	nodular	mut	anti-PD1	1	Brain	PR
14		m, 65	2	naevoid	mut	anti-PD1 + anti-CTLA4	4	Soft tissue, Lung, Lymph nodes, Brain	PR
15	Non-Responders	m, 79	2	LMM^6^	wt	anti-PD1	2	Lymph nodes, Liver	SD^*^
16		f, 52	2	SSM	mut	anti-CTLA4	5	Soft tissue, Lung, Liver, Mesenterium, Brain	PD
17		m, 68	2	SSM	wt	anti-PD1	8	Soft tissue, Lymph nodes, Lung, Suprarenal gland, Liver, Intestinum, Bone, Brain	PD
18		f, 58	3	nodular	mut	anti-PD1	1	Brain	PD
19		m, 85	3	nodular	wt	anti-PD1	1	Brain	PD
20		m, 60	3	nodular	wt	anti-PD1 + anti-CTLA4	3	Lymph nodes, Lung, Liver	PD
21		m, 75	n.a.	desmoplastic	wt	anti-PD1	2	Lymph nodes, Liver	PD

* pseudoprogression, ^1^male, ^2^female, ^3^years, ^4^not applicable, ^5^superficial spreading melanoma, ^6^lentigo maligna melanoma, ^7^wild type, ^8^V600E mutation, ^9^anti-programmed-cell-death-protein-1, ^10^anti-cytotoxic-T-lymphocyte-associated-protein-4, ^11^*CR* Complete Remission, *PR* Partial Remission, *SD* Stable Disease, *PD* Progressive Disease

**Fig. 1:**
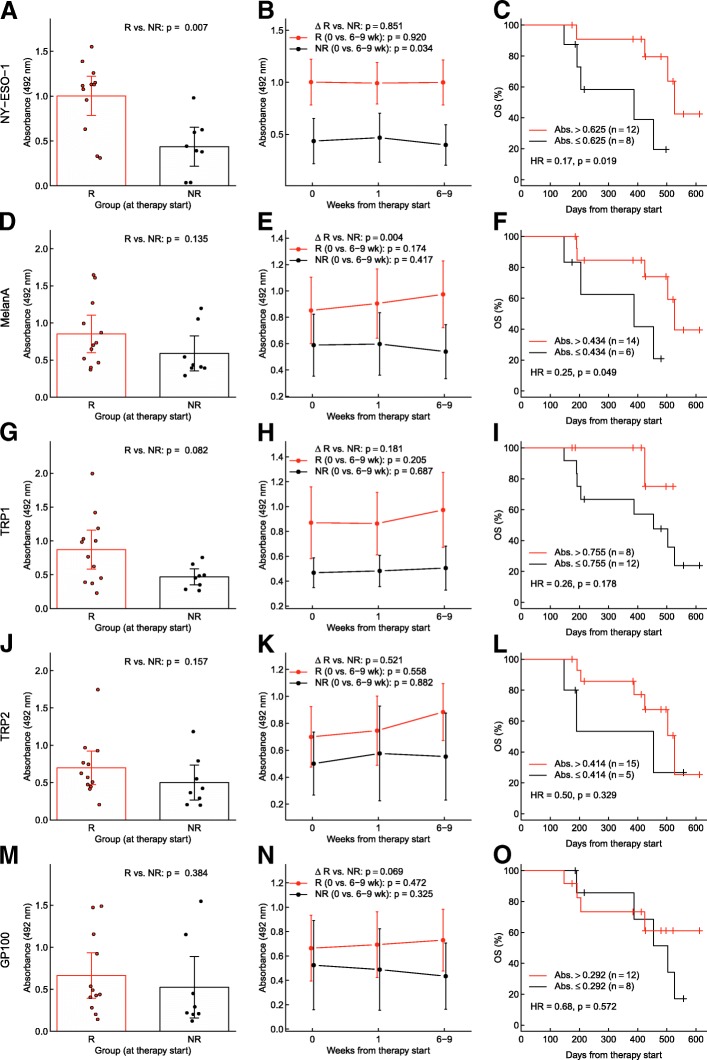
Melanoma-specific antibody kinetics and overall survival in cohort 1. Antibody levels and kinetics in the sera of responders (R), non-responders (NR): Anti-NY-ESO-1 (**a, b**), anti-MelanA/MART1 (**d, e**), anti-TRP1/TYRP1 (**g, h**), anti-TRP2/TYRP2 (**j, k**), anti-gp100 (**m, n**). **a, d, g, j, m**: Antibody levels before treatment start. Differences between responders and non-responders were tested with Wilcoxon rank-sum tests. Bars represent means and 95% CI, and circles show data from individual patients. **b, e, h, k, n**: Differences between the three visits (i.e. change during checkpoint inhibitor therapy) were tested with Friedman tests for each patient group. Changes (Δ) in IgG levels from treatment start to the visit after 6–9 weeks were compared between responders and non-responders with Wilcoxon ranks sum tests; *p*-values for this test are given above those for every group. Bars represent means and 95% CI. **c, f, i, l, o**: Kaplan-Meier curves showing overall survival (OS) of patients with high vs. low antibody levels at therapy start. Grouping criteria (cutpoints) are given in graphs. Hazard ratios (HR) for high vs. low antibody levels are provided with *p*-values from log-rank tests

We first determined if responders and non-responders differed in their specific antibody levels before start of CI therapy, and whether the levels changed over the course of therapy. In cohort one we found that antigen specific antibody absorbances were higher in responders (R) compared to non-responders (NR), see Fig. 1a, d, g, j, m. These differences were most pronounced and statistically significant for NY-ESO-1 (R vs. NR: *p =* 0.007).

Over the course of therapy specific antibody levels increased or stayed unchanged in the responder group, while they decreased in the non-responder group (Fig. 1b, e, h, k, n). However, these trends and group differences were not of statistical significance.

In both cohorts, overall and progression free survival were significantly longer in responders according to RECIST 1.1 (Additional file 2: Figure S1). Patients were divided into groups showing high or low specific antibody levels. Receiver operating curves (ROC) analysis was used to determine the optimal threshold for the antibody level against each antigen maximizing the sum of sensitivity and specificity for the prediction of the radiological responses. These groups were then tested for OS and PFS. Interestingly, patients with higher antibody levels for NY-ESO-1 and MelanA/MART1 at baseline had a significantly longer OS (anti-NY-ESO-1: *HR* = 0.17, *p* = 0.019; anti-MelanA/MART1: *HR* = 0.25, *p* = 0.049) (Fig. 1 c, f, i, l, o). Patients with higher absorbance levels also had a significantly longer PFS (anti-NY-ESO-1: *HR* = 0.31, *p =* 0.043; anti-TRP1/TYRP1: *HR* = 0.29, *p* = 0.050, anti-gp100: *HR* = 0.27, *p* = 0.022) (Additional file 2: Figure S2).

In the control (NSCLC) group, no significant differences in antibody levels were found between NSCLC responders and non-responders, both before start of CI therapy and after 6–9 weeks of treatment (Additional file 2: Figure S3A-E).

**Fig. 2:**
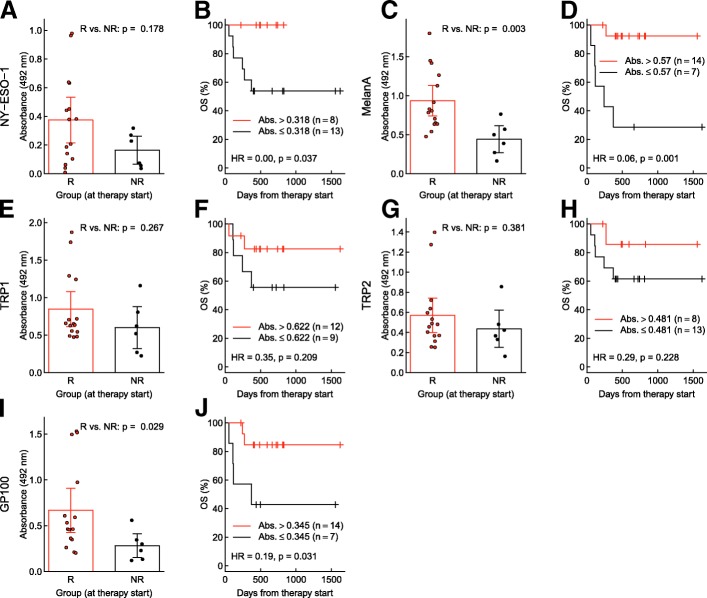
Melanoma-specific antibody responses and overall survival in cohort 2. **a, b:** Anti-NY-ESO-1, **c, d:** anti-MelanA/MART1, **e, f:** anti-TRP1/TYRP1, **g, h:** anti-TRP2/TYRP2, **i, j:** anti-gp100. **a, c, e, g, i**: Differences between responders (R) and non-responders (NR) were tested with Wilcoxon rank-sum tests. Bars represent means and 95% CI, and circles show data from individual patients. **b, d, f, h, j**: Kaplan-Meier curves showing overall survival (OS) of patients with high vs. low antibody levels at therapy start. Grouping criteria (cutpoints) are given in graphs. Hazard ratios (HR) for high vs. low antibody levels are provided with *p*-values from log-rank tests

In cohort two, which was independent of cohort one, significantly higher levels of specific antibodies against MelanA/MART1 (*p =* 0.003) and gp100 (*p =* 0.029) were detected at baseline in the responder group (Fig. 2c, i). In addition, antibodies against NY-ESO-1, TRP1/TYPR1 and TRP2/TYRP2 showed a trend towards higher levels in responders (Fig. 2a, e, g). Similar to cohort one, patients with higher IgG absorbances for anti-NY-ESO-1 (*HR* = 0.00, *p* = 0.037), anti-MelanA/MART1 (*HR* = 0.06, *p* = 0.001) and anti-gp100 (*HR* = 0.19, *p* = 0.031) showed significantly longer OS compared to patients below the threshold (Fig. 2b, d, f, h, j). High IgG levels against MelanA/MART1 and gp100 levels resulted in a significantly longer PFS (anti-MelanA/MART1: *HR* = 0.18, *p* = 0.011, anti-gp100: *HR* = 0.19, *p* = 0.014) (Additional file 2: Figure S4A-E). An overall assessment of total serum IgG was carried out to check the patients’ immune status at a more global scale. Total IgG did not differ significantly at baseline and during treatment between responders and non-responders. (Additional file 2: Figure S5A, B).

Furthermore, we measured anti-EBNA-1 IgG and anti-VZV IgG in the patients’ sera. In contrast to MDA and C/T antigen specific antibodies, anti-EBNA-1 IgG and anti-VZV IgG antibody titers neither differed between responders and non-responders, nor did anti-EBNA-1 IgG titers change during the course of therapy (Additional file 2: Figure S6A, B). This indicates that pre-existing antibodies against irrelevant proteins are not influenced by CIs.

**Fig. 3:**
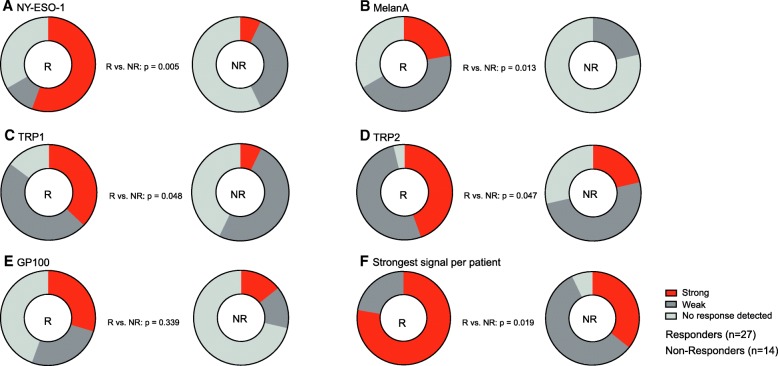
Specific antibodies against melanoma-specific self-antigens pooled in strong, weak and negative signals after merging the two cohorts. **a** Anti-NY-ESO-1, **b** anti-MelanA/MART1, **c** anti-TRP1/TYRP1, **d** anti-TRP2/TYRP2, **e** anti-gp100 ELISA absorbance signals were classified in “strong”, “weak” and “no response detected” by taking the mean value of the control group of cohort 1 as cutpoint for a weak positive signal and its double as cutpoint for a strong positive signal. Differences between responder (R) and non-responders (NR) were tested with Fisher’s exact test. **f** In addition, patients were classified according to the strongest signal obtained with any of the five antigens

In order to compare responders and non-responders from both cohorts, the results from the two cohorts were merged and then classified into patients with “no response detected”, “weak” and “strong” antibody responses for each of the antigens. Responders had significantly more “strong” absorbance signals for NY-ESO-1, MelanA/MART1, TRP1/TYRP1 and TRP2/TYRP2 (Fig. 3a-d). In addition, we classified the patients according to whether they showed a strong signal to any of the five antigens. This also showed a significant association between stronger signal and response to therapy (*p* = 0.019) (Fig. 3e).

Immunohistochemistry was performed with the available tumor tissue with correlative analysis of IgG levels specific for gp100 and MelanA/MART1. There was no significant correlation between serum IgG levels of gp100 and its antigen expression in the tumor (r [9] = − .2974, *p* = 0.4370) or MelanA/MART1 and its antigen expression (r [9] = − .3167, *p* = 0.4101) before the start of treatment, which further supports the independent character of the association between the presence of these antibodies in the serum of metastatic melanoma patients and their better overall survival (Additional file 2: Figure S7A-F).

## Discussion

In this study, we addressed the role of pre-existing MDA and C/T antigen specific antibodies as potential biomarkers for CI response and survival in patients suffering from metastatic melanoma. To our knowledge, we demonstrate for the first time in two independent melanoma patient cohorts that responders to CI therapy have higher pre-treatment levels of antibodies specific for MDA (TRP1/TYRP1, TRP2/TYRP2, gp100, MelanA/MART1) and the C/T antigen NY-ESO-1. To further speculate on the function of the measured antibodies, we determined the four IgG subclasses of the specific antibodies in serum samples of cohort one. These preliminary experiments show interesting results: NY-ESO-1, TRP1/TYRP1 and TRP2/TYRP2 specific antibodies consisted of several subclasses. MelanA/MART-1 specific antibodies consisted mainly of IgG1, gp100 mainly of IgG2 subclass. Interestingly, none of these specific antibodies were of IgG4 subclass (Additional file 2: Figure S8).

Our findings suggest that these antibodies may be a predictive surrogate marker for response to CI therapy. This is in line with a recent study showing that NY-ESO-1 seropositive melanoma patients had a favorable response to ipilimumab [49]. Of note, total IgG and IgG titers against irrelevant viral antigens EBV and VZV were similar in responders and non-responder.

The vast majority of such antibodies are directed against tumor cell internal epitopes and are therefore not involved in the anti-tumor immune response but rather a surrogate marker for an ongoing immune response. However, a few antibodies have been shown to indeed recognize tumor cell surface epitopes; the most prominent example is the monoclonal antibody TA99 specific for TRP1/TYRP1 [50]. Unfortunately, the efficacy of monotherapy with the tumor-antigen specific antibody IMC-20D7S (Anti-TRP1/TYRP1 monoclonal antibody) was limited in clinical trials, though it was well tolerated [51]. Nevertheless, targeting tumors with antibodies in combination therapies can result in significant synergies [52, 53]. The importance of antibodies is not only true for melanoma as a group has recently shown in prostate cancer that clinical responders to CTLA4-blockade and granulocyte macrophage colony-stimulating factor (GM-CSF) developed enhanced antibody responses to a higher number of antigens than non-responders and that pre-existing antibodies to these antigens were more likely to be present in the clinical responders compared to non-responders [54].

The interaction between B and T cells may be particularly important if the immune response is directed against true self antigens as a recent paper has demonstrated that self-reactive T cells in multiple sclerosis were only able to penetrate into the brain tissue when they had help by antigen-experienced B cells [55].

It will also be important to determine the role of antibodies in tumors with a high mutational load. The interplay between antibodies and T cells may be less important if the T cells recognize neoantigens. Alternatively, B cell responses may occur and play potential roles when high mutational load-tumors express B cell neoepitopes.

A strength of our study is the prospective character of cohort 1, which ensures a complete data set. The main findings were then confirmed in an independent second cohort. Furthermore, all ELISAs were carried out in duplicates and in a blinded fashion.

However, there are also several limitations. Firstly, patient numbers are low; however, the statistically significant results and the prospective character of the study strengthen our data. Secondly, the follow-up time is limited, but sufficient to make a statement about response and PFS. For long-term survival additional follow-up data is required.

## Conclusions

Our study showed for the first time that high levels of melanoma-associated antibodies are independently correlated with response to CI treatment and prolonged PFS and OS. These antibodies may therefore be useful as potential new biomarkers in patients with metastatic melanoma.

Tumor-specific antibodies directed against MDA (TRP1/TYRP1, TRP2/TYRP2, gp100, MelanA/MART1) and against the C/T antigen NY-ESO-1 are candidate biomarkers that may complement patient assessment in association with PD-L1 status and/or TILs, with the aim to predict outcomes of CI treatment in patients with metastatic melanoma. Finally, novel combination therapies may be re-considered with recombinant tumor-specific antibodies targeting those B cell epitopes that are displayed on the tumor cell surface such as TRP1/TYRP1.

## Additional files

Additional file 1: **Table S1.** ELISA setup. (DOCX 19 kb)

Additional file 2: **Figure S1.** Overall and progression free survival in cohort 1 and cohort 2. **Figure S2.** Progression free survival in cohort 1. **Figure S3.** Melanoma-associated antibodies in the NSCLC control cohort. **Figure S4.** Progression free survival in cohort 2. **Figure S5.** Total IgG before start with checkpoint inhibitor and total IgG kinetics. **Figure S6.** Anti-EBV antibody response during treatment with checkpoint inhibitor. **Figure S7.** gp100 and MelanA/MART1 specific antibodies and corresponding antigen expression in tumor tissue. **Figure S8.** IgG subclasses of melanoma-associated antibodies. (PDF 974 kb)

## Abbreviations

ADCC: Antibody-dependent cellular cytotoxicity
C: Control cohort
C/T: Cancer/testis
CIs: Checkpoint inhibitors
CLIA: Chemiluminescence Immunoassay
CR: Complete remission
CRPC: Castration resistant prostate cancer
CT: Computed tomography
CTLA4: Cytotoxic-T-lymphocyte-associated-protein-4
CV: Coefficient of variations
ELISA: Enzyme-linked immunosorbent assay
GM-CSF: Granulocyte macrophage colony-stimulating factor
gp100: Glycoprotein 100
HPF: High power field (micrograph acquired with an 40x objective/400x magnification)
irAEs: Immune-related adverse events
LDH: Lactate dehydrogenase
MDA: Melanocyte differentiation antigen
NR: Non-responders
NSCLC: Non-small-cell-lung-cancer
OS: Overall survival
PD: progressive disease
PD1: programmed-cell-death-protein-1
PD-L1: programmed-cell-death-1-ligand-1
PFS: progression free survival
PR: partial remission
R: Responders
ROC: Receiver operating curves
SD: Stable disease
TILs: Tumor-infiltrating lymphocytes
TRP1/TYRP1: Tyrosinase-related proteins 1
TRP2/TYRP2: Tyrosine-related proteins 2
URPP: University Research Priority Program
VZV: Varicella zoster virus
