# Supplementary material for: Antibodies as biomarker candidates for response and survival to checkpoint inhibitors in melanoma patients
Source: J Immunother Cancer. 2019 Feb 20;7:50. doi: 10.1186/s40425-019-0523-2 (PMC6383238; doi:10.1186/s40425-019-0523-2)
Supplement: Supplementary file 1 — Table S1. ELISA setup. (DOCX 19 kb) [file 40425_2019_523_MOESM1_ESM.docx]

**Table S1**

| **Coating antigen** | **amount/well (µg)** | **tested amounts/well (µg)** | **serum dilution** | **Tested serum dilutions** | **source** |
| --- | --- | --- | --- | --- | --- |
| NY-ESO-1 | 0.0625 | 0.5,0.25,0.125,0.0625,0.0312 | 1:1280 | 1:80, 1:160, 1:320, 1:640, 1:1280 | yeast |
| MelanA | 0.25 | 0.5, 0.25, 0.125, 0.0625 | 1:10 | 1:5, 1:10, 1:20, 1:40, 1:80 | wheat |
| TRP1 | 0.25 | 0.5, 0.25, 0.125, 0.0625 | 1:10 | 1:5, 1:10, 1:20, 1:40, 1:80 | wheat |
| TRP2 | 0.5 | 0.5, 0.25, 0.125, 0.0625 | 1:20 | 1:5, 1:10, 1:20, 1:40, 1:80 | wheat |
| gp100 | 0.25 | 0.5, 0.25, 0.125, 0.0625 | 1:10 | 1:5, 1:10, 1:20, 1:40, 1:80 | wheat |
