# Supplementary material for: Antibodies as biomarker candidates for response and survival to checkpoint inhibitors in melanoma patients
Source: J Immunother Cancer. 2019 Feb 20;7:50. doi: 10.1186/s40425-019-0523-2 (PMC6383238; doi:10.1186/s40425-019-0523-2)
Supplement: Supplementary file 2 — Figure S1. Overall and progression free survival in cohort 1 and cohort 2. Figure S2. Progression free survival in cohort 1. Figure S3. Melanoma-associated antibodies in the NSCLC control cohort. Figure S4. Progression free survival in cohort 2. Figure S5. Total IgG before start with checkpoint inhibitor and total IgG kinetics. Figure S6. Anti-EBV antibody response during treatment with checkpoint inhibitor. Figure S7. gp100 and MelanA/MART1 specific antibodies and corresponding antigen expression in tumor tissue. Figure S8. IgG subclasses of melanoma-associated antibodies. (PDF 974 kb) [file 40425_2019_523_MOESM2_ESM.pdf]

Supplementary Figure 1

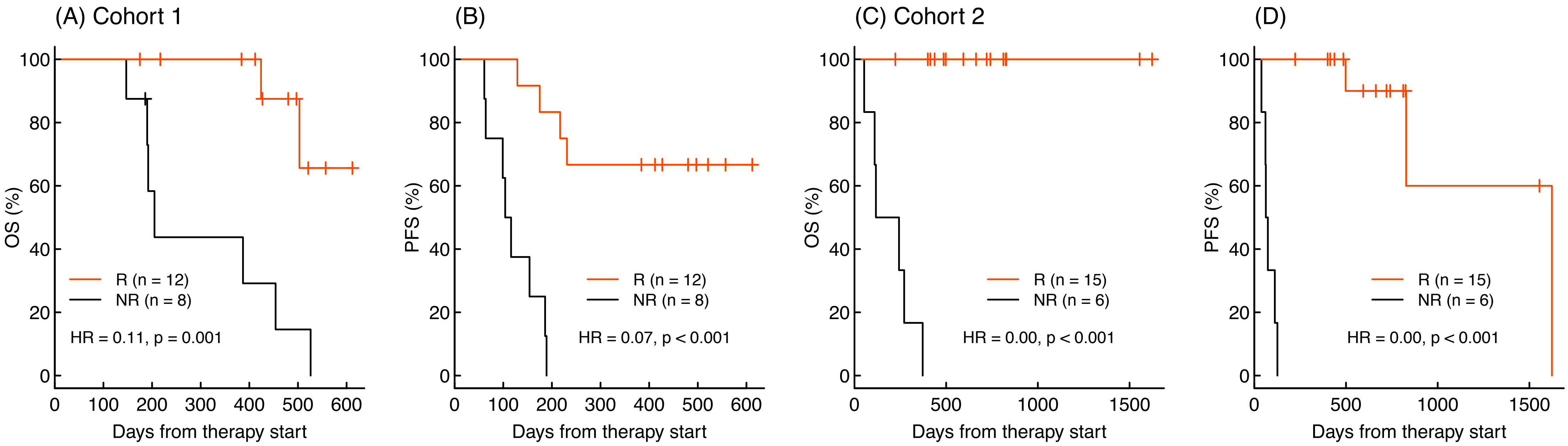

Supplementary Figure 2

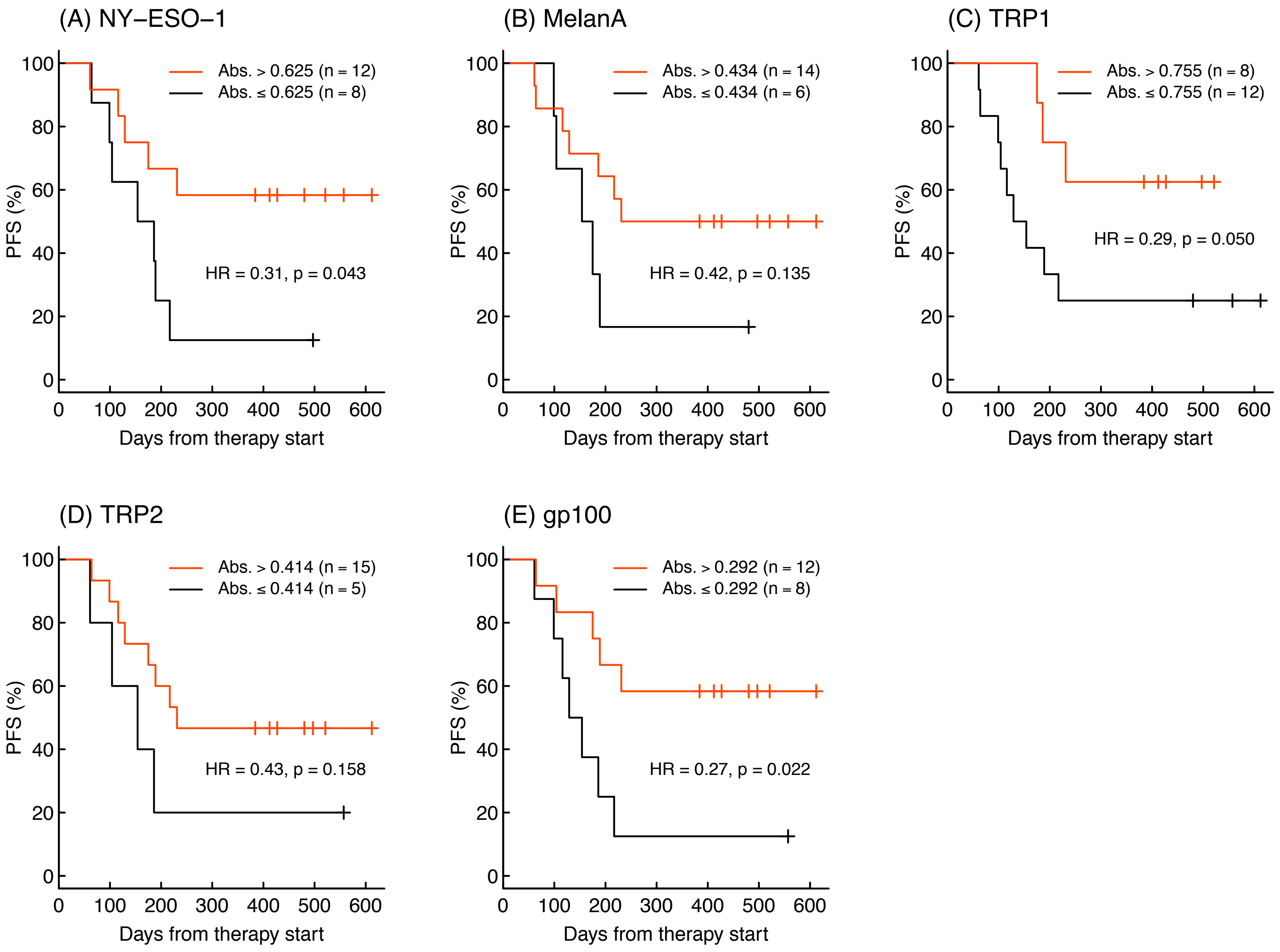

Supplementary Figure 3

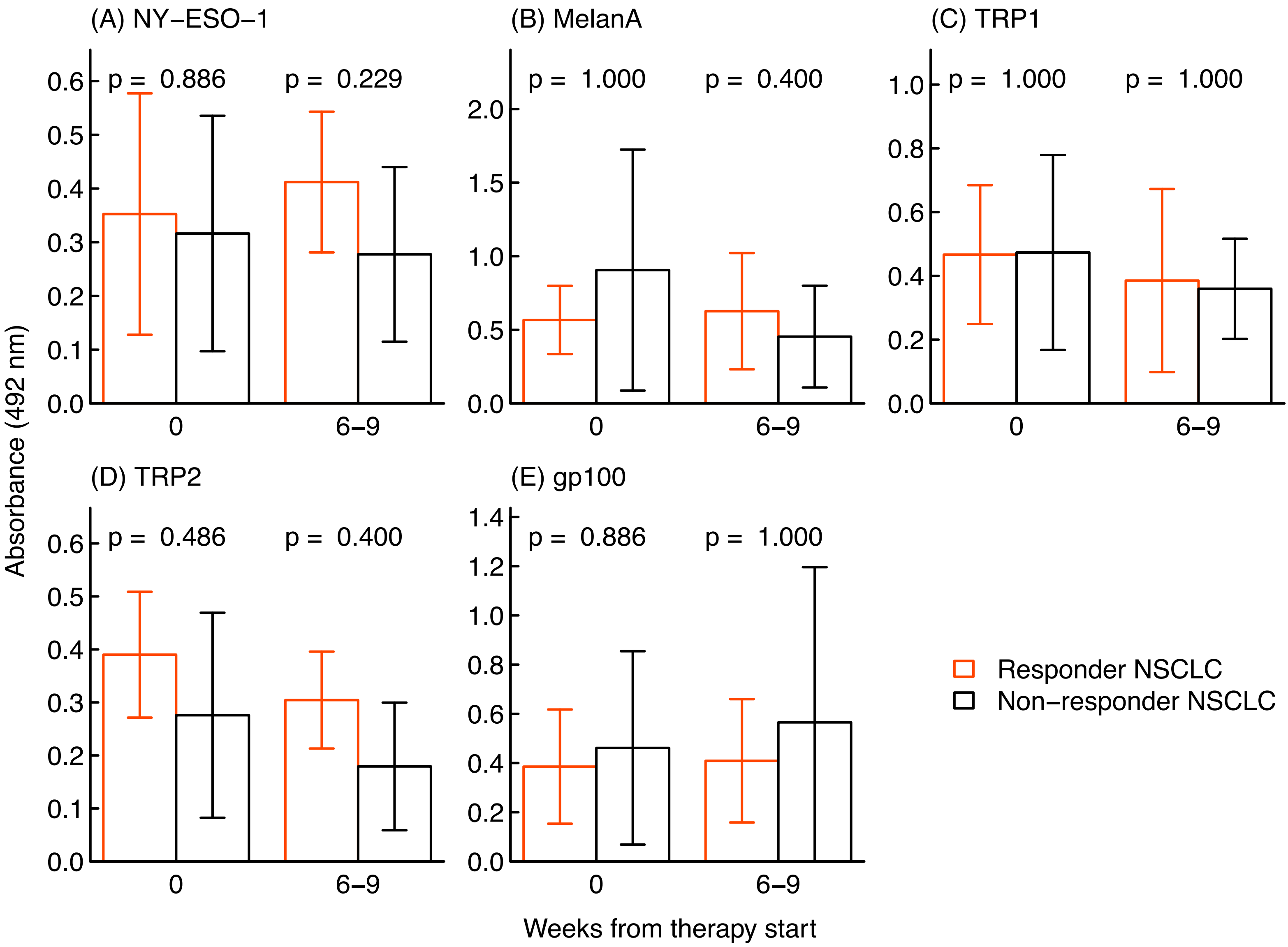

Supplementary Figure 4

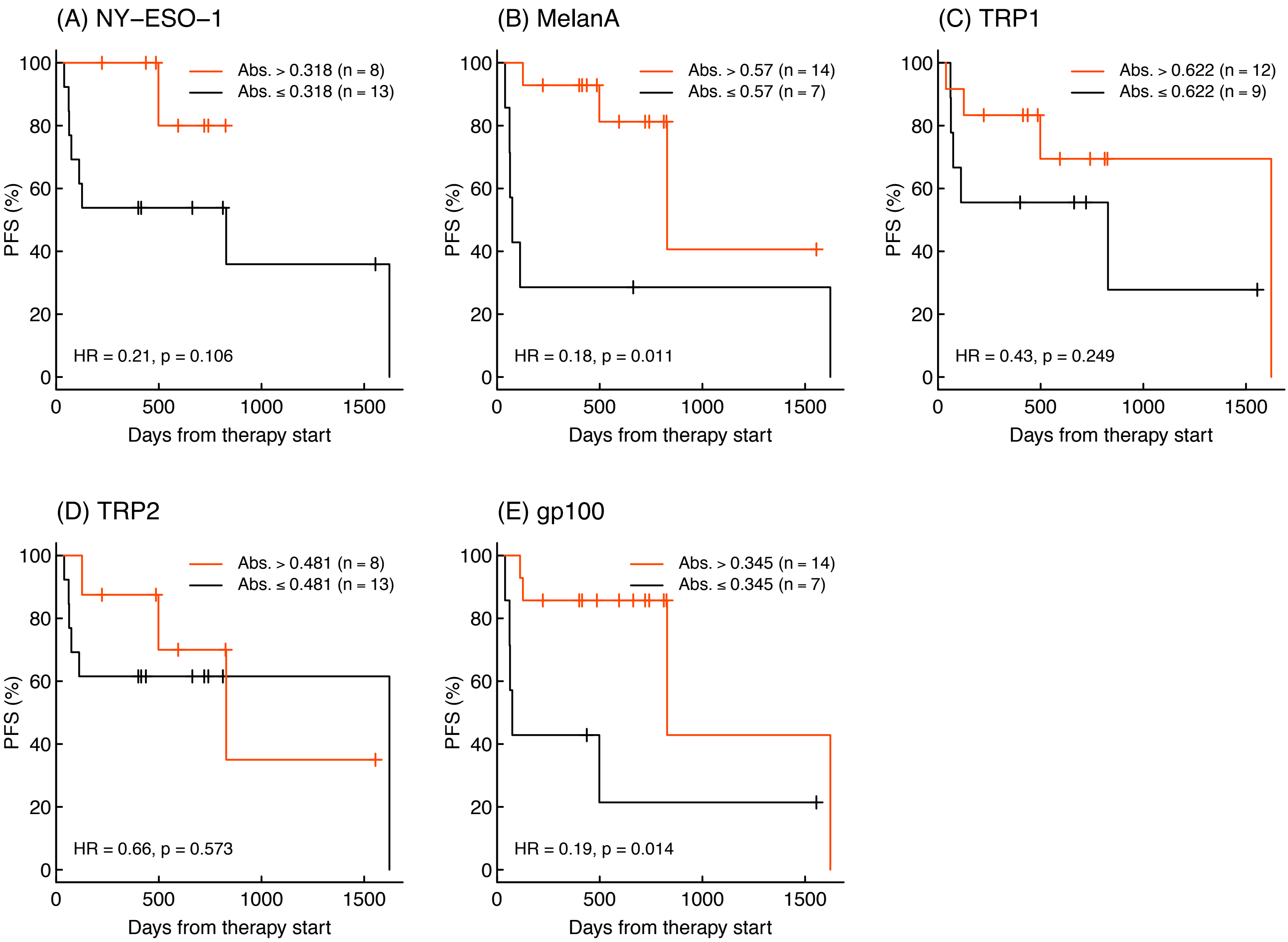

Supplementary Figure 5

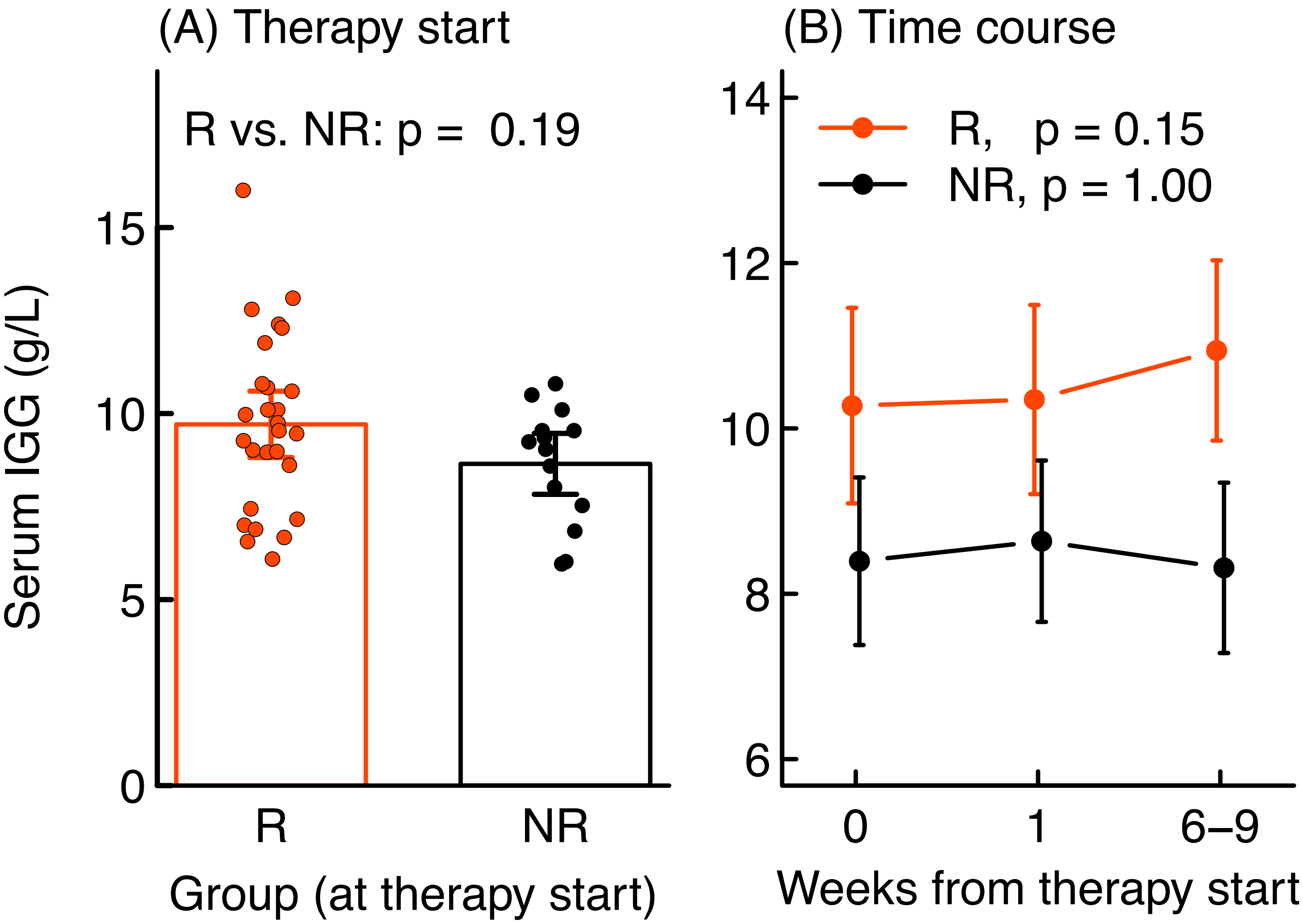

Supplementary Figure 6

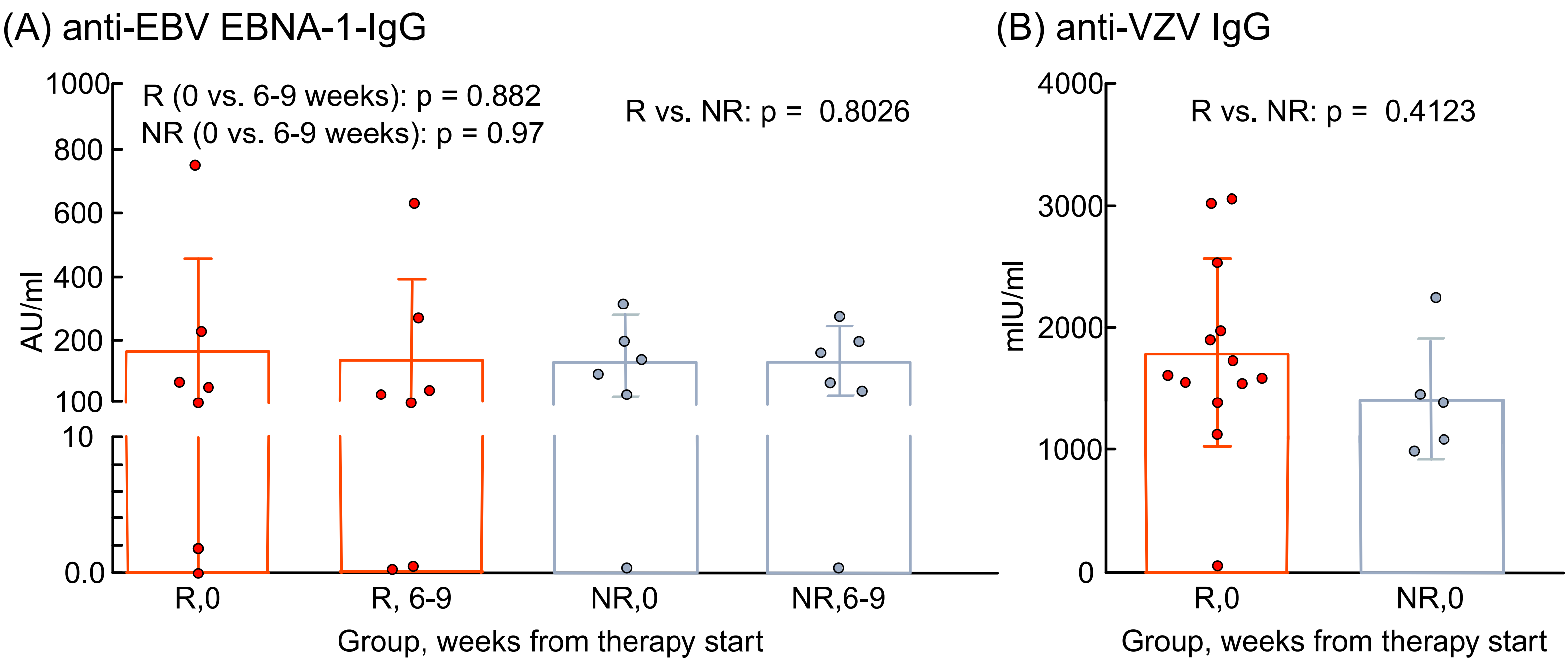

Supplementary Figure 7

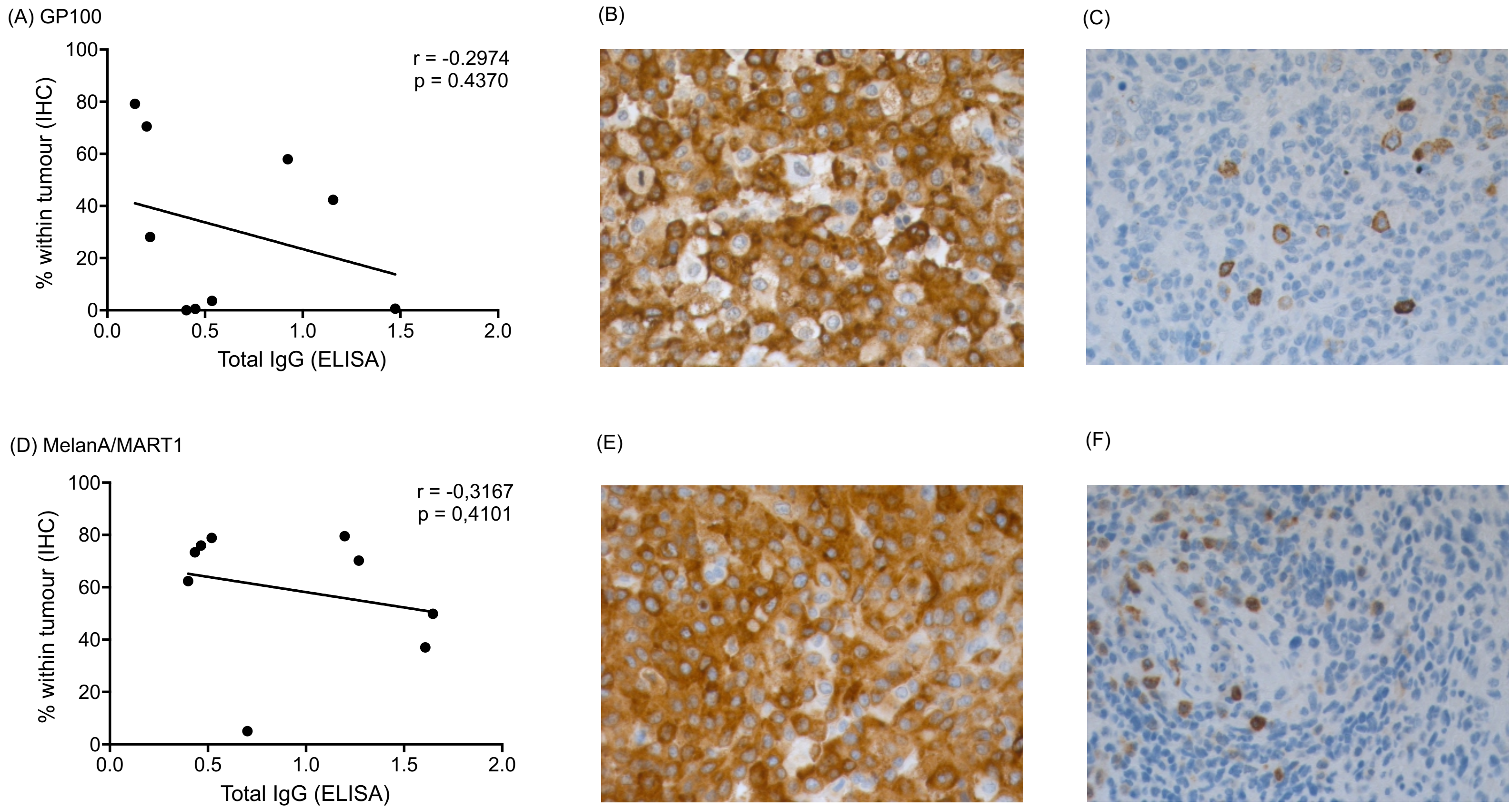

Supplementary Figure 8

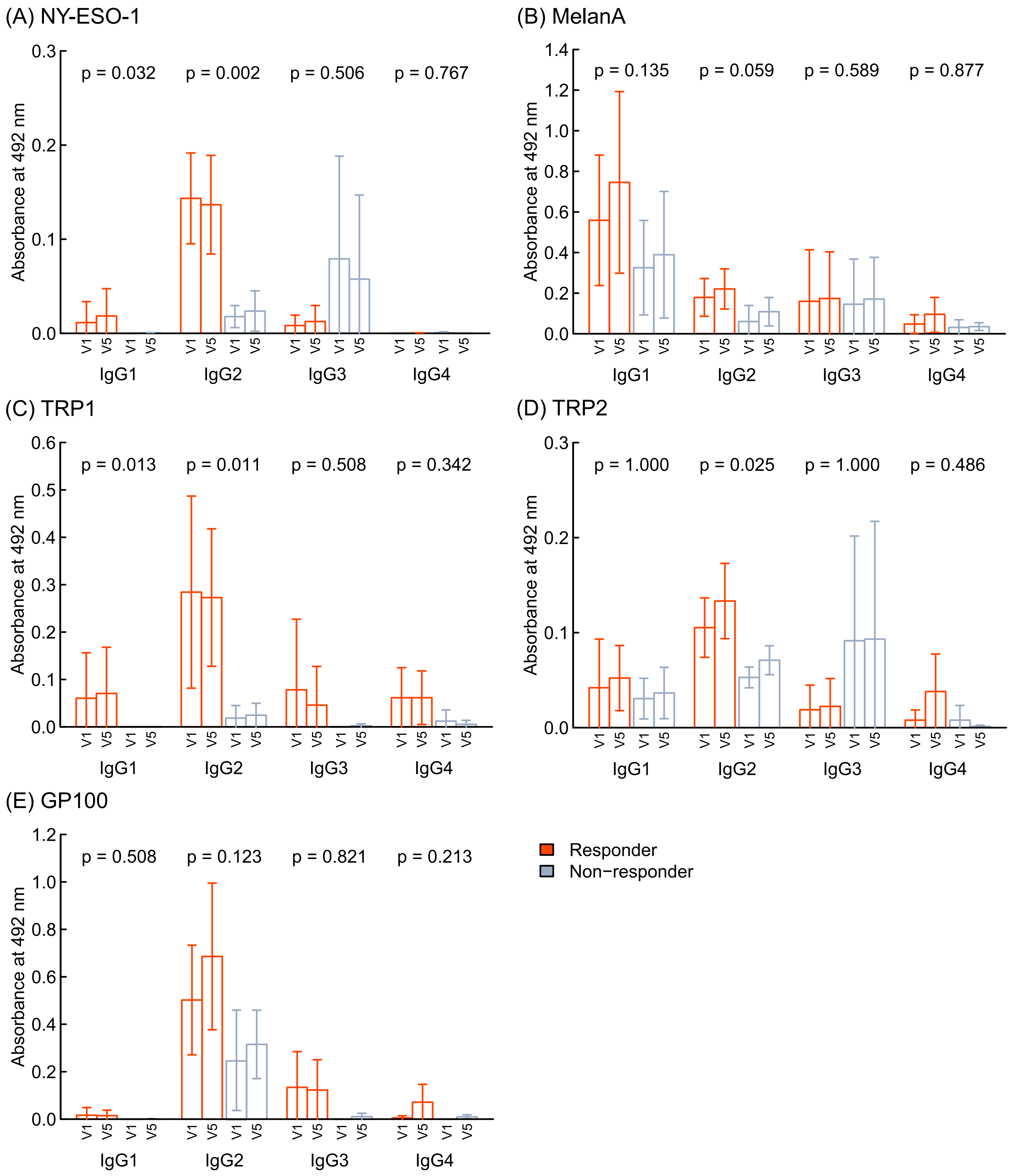

Figure Legends Supplementary Figures

Supplementary Figure 1: Overall and progression free survival in cohort 1 and cohort 2.
Kaplan-Meier curves showing overall survival (OS) (A, C) and progression free survival (PFS) (B, D) for responders (R) and non-responders (NR) in cohort 1 (A, B) and cohort 2 (C, D). Hazard ratios (HR) for antibody levels of R and NR are provided with *p*-values from log-rank tests.

Supplementary Figure 2: Progression free survival in cohort 1.
A-E: Kaplan-Meier curves showing progression free survival (PFS) of patients with high vs. low antibody levels at therapy start. Grouping criteria (cutpoints) are given in graphs. Hazard ratios (HR) for high vs. low antibody levels are provided with *p*-values from log-rank tests.

Supplementary Figure 3: Melanoma-associated antibodies in the NSCLC control cohort.
Antibody levels before treatment start and after 6-9 weeks of treatment in the sera of responders (R) and non-responders (NR) of the NSCLC control (C) patients from cohort 1: Anti-NY-ESO-1 (A), anti-MelanA/MART1 (B), anti- TRP1/TYRP1 (C), anti-TRP2/TYRP2 (D), anti-gp100 (E). Differences between responders and non-responders were tested with Wilcoxon rank-sum tests. Bars represent means and 95% CI.

Supplementary Figure 4: Progression free survival in cohort 2.
A-E: Kaplan-Meier curves showing progression free survival (PFS) of patients with high vs. low antibody levels at therapy start. Grouping criteria (cutpoints) are given in graphs. Hazard ratios (HR) for high vs. low antibody levels are provided with *p*-values from log-rank tests.

Supplementary Figure 5: Total IgG before start with checkpoint inhibitor and total IgG kinetics.
(A) Antibody levels of total IgG before treatment start in the sera of responders (R) and non-responders (NR) of cohort 1 and 2 (combined). Differences between responders and non-responders were tested with Wilcoxon rank-sum tests. Bars represent means and 95% CI. (B) Total IgG antibody level kinetics in the sera of responders (R) and non-responders (NR) from cohort 1. Differences between the three visits (i.e. change during checkpoint inhibitor therapy) were tested with Friedman tests for each patient group; *p*-values are given above those for every group. Bars represent means and 95% CI.

Supplementary Figure 6: Anti-EBV antibody response during treatment with checkpoint inhibitor.
Bars showing differences in levels of anti-EBNA-1-IgG from 7 responders (R) and 6 non-responders (NR) from cohort 1 during checkpoint inhibitor therapy (A) and in levels of anti-VZV-IgG from 13 R and 5 NR from cohort 1 and 2 before checkpoint inhibitor therapy (B). Bars represent means and 95% CI, *p*-values are given from paired t-tests and circles show data from individual patients.

Supplementary Figure 7: gp100 and MelanA/MART1 specific antibodies and corresponding antigen expression in tumor tissue.
Correlative analysis to examine the relation between IgG levels specific for gp100 (A) and MelanA/MART1 (D) in serum and their corresponding antigen expression in tumor tissue revealed no correlation in either of the cases before the start of the treatment (Pearson correlation, $r(9) = -.2974$, $p = .4370$ and Spearman correlation $r(9) = -.3167$, $p = .4101$ respectively). Representative micrographs from tumors with high (B) and low (C) gp100 expression, as well as high (E) and low (F) MelanA/MART1 expression before start of treatment.

Supplementary Figure 8: IgG subclasses of melanoma-associated antibodies. Differences between responders and non-responders in levels of melanoma-specific antibodies (IgG subclasses 1 to 4) in cohort 1 during checkpoint inhibitor therapy. Bars represent means and 95% CI per patient group and visit. Differences between patient groups were tested with Wilcoxon rank-sum tests based on mean values of visits 1 and 5 for each patient; *p*-values are given above each bar group.

Supplementary Table 1:
ELISA setup.
